# Identification of *Echinococcus granulosus* strains using polymerase chain reaction–restriction fragment length polymorphism amongst livestock in Moroto district, Uganda

**DOI:** 10.4102/ojvr.v83i1.1068

**Published:** 2016-07-29

**Authors:** Martin Chamai, Leonard Omadang, Jospeh Erume, Michael Ocaido, Peter Oba, Emmanuel Othieno, Straton Bonaventure, Annah Kitibwa

**Affiliations:** 1 Department of Biomolecular Resources and Biolaboratory Sciences, College of Veterinary Medicine, Animal Resources and Biosecurity, Makerere University, Uganda; 2 Department of Pathology, College of Health Sciences, Makerere University, Uganda

## Abstract

A descriptive study was conducted to identify the different strains of *Echinococcus granulosus* occurring in livestock in Moroto district, Uganda. *Echinococcus* cysts from 104 domestic animals, including cattle, sheep, goats and camels, were taken and examined by microscopy, polymerase chain reaction with restriction fragment length polymorphism and Sanger DNA sequencing. *Echinococcus granulosus* genotypes or strains were identified through use of Bioinformatics tools: BioEdit, BLAST and MEGA6. The major finding of this study was the existence of a limited number of *E. granulosus* genotypes from cattle, goats, sheep and camels. The most predominant genotype was G1 (96.05%), corresponding to the common sheep strain. To a limited extent (3.95%), the study revealed the existence of *Echinococcus canadensis* G6/7 in three (*n* = 3) of the *E. granulosus*–positive samples. No other strains of *E. granulosus* were identified. It was concluded that the common sheep strain of *Echinococcus sensu stricto* and G6/7 of *E. canadensis* were responsible for echinococcal disease in Moroto district, Uganda.

## Introduction

Echinococcosis in humans is one of the neglected zoonotic diseases. It is caused by metacestodes of the genus *Echinococcus,* with the main definitive host as the dog (Eckert *et al*. 2001). This genus includes small tapeworms of carnivores with larval stages known as hydatid cysts or metacestodes that proliferate in various organs of the intermediate hosts, including humans. The genus *Echinococcus* included four morphologically indistinct species (*Echinococcus granulosus, Echinococcus multilocularis, Echinococcus oligarthrus* and *Echinococcus vogeli*) until recently, when *Echinococcus shiquicus* and *Echinococcus felidis* were added as the fifth and sixth species (Huttner *et al*. 2008; Xiao *et al*. 2005).

Currently, there are four forms of hydatidosis that have been deemed taxonomically valid (Soulsby 1982). These include cystic echinococcosis (CE) (*E. granulosus*), alveolar echinococcosis (*E. multilocularis*) and polycystic echinococcosis (*E. vogeli* and *E. oligarthrus*). Of these, the most common form is cystic, followed by alveolar, echinococcosis. Alveolar echinococcosis is more severe and fatal than CE but sporadic and more difficult to treat because of its characteristic multiple budding and infiltration into organs, including the brain (Eckert *et al*. 2001).

CE is prevalent in sub-Saharan Africa (Macpherson & Wachira 1997) and is caused by the metacestode of the dog tapeworm *E. granulosus*. The disease is currently considered an emerging zoonotic disease worldwide (Eckert *et al*. 2001), with an estimated global burden of over 1 million disability-adjusted life-years lost (Budke, Deplazes & Torgerson 2006). This is almost equal to the burden caused by African trypanosomiasis and schistosomiasis (Budke, Deplazes & Torgerson 2006). In China, for example, CE has been worse, with between 60 000 and 1 300 000 people affected, with children accounting for one-third (Craig 2005).

CE transmission is more intense in remote pastoral communities or regions where veterinary services are poor and where dogs have easy access to animal carcasses and offal. It has been suggested that some of the risk factors responsible for human CE include livestock raising, pastoral life, gender, ethnicity, dog ownership, poor hygiene and low socio-economic status (Dowling, Abo-Shehada & Torgerson 2000; Larrieu *et al*. 2002).

Economically, CE poses a threat to animal farming (Thompson 2008), leading to losses because of condemnation of edible organs, reduced meat and milk production, decreased hide and fleece value and decrease in fecundity (Polydorou 1981; Romazanov 1983). The public health concern is more critical, and as yet, there are no control measures in place. In eastern Africa, areas with high prevalence of human CE are said to be focally distributed in Kenya, northern Tanzania and South Sudan, with prevalence of at least 6% amongst nomadic populations (Macpherson *et al*. 1989). There is a significant public health problem amongst pastoralists in the region, especially the Turkana and Maasai communities in Kenya (French & Nelson 1982). One of the highest reported incidences of human CE (220/100 000) in the world was in the Turkana district of north-western Kenya (French & Nelson 1982). Likewise, in South Sudan, human CE is frequent in the extreme south-east of the country at the border with Kenya, where prevalence stands at 2% – 3.5% as examined by ultrasound scans (Magambo *et al*. 1996; Magambo, Zeyhle & Wachira 1998). In Uganda, Inangolet *et al*. (2010) conducted a study to establish the prevalence of *E. granulosus* amongst dogs in Karamoja and found the prevalence to be 66.3%; however, no information on genotypes was provided. This information provides the background to the study.

## Materials and methods

### Study area

**FIGURE 1:**
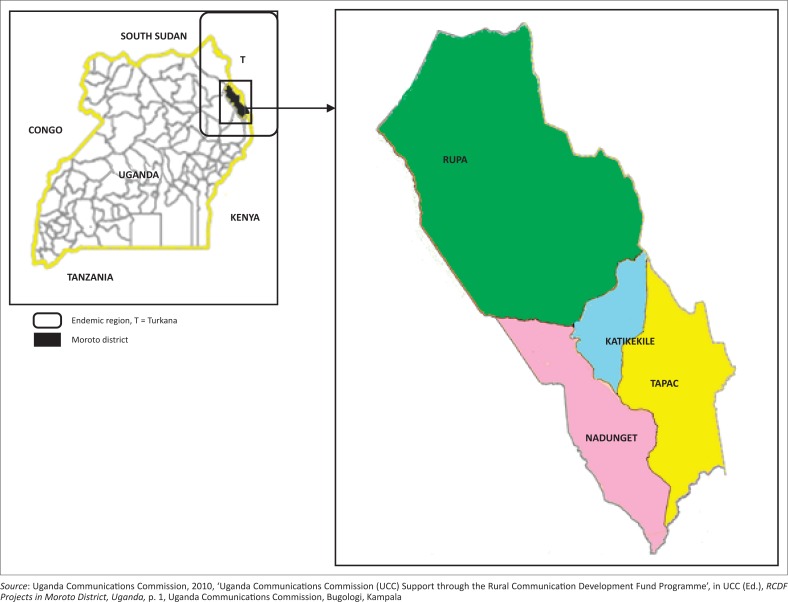
Map of Uganda showing one of echinococcal endemic regions (circled pattern) and administrative sub-counties of Moroto district.

The study was conducted between February 2013 and August 2014 in Moroto district, sub-region of Karamoja. Karamoja sub-region is located in north-eastern Uganda and comprises seven districts, namely Abim, Amudat, Kaabong, Kotido, Moroto, Nakapiripirit and Napak. The coordinates of the town (Moroto) are 2°31’48.0”N, 34°40’12.0”E (Latitude: 2.5300; Longitude: 34.6700). It is bordered in the North by South Sudan, in East by Turkana region and in the West by Pokot district of Kenya. Moroto district was chosen because of high incidences of CE (Inangolet *et al*. 2010) and its proximity to Turkana (Figure 1), which once had the world’s highest incidences (Macpherson 1998; Macpherson *et al*. 1989).

### Study design

This was a descriptive study where samples were obtained from livestock at Moroto Municipal Council abattoir. Details of the species of slaughter livestock (Table 1) were recorded including numbers of livestock. Upon inspection, infected organs were isolated and stored until a large sample size (*n* ≥ 20 samples) was reached. Samples were collected every 3 months and transported to Makerere University Molecular Biology Laboratory for testing.

**TABLE 1:** Areas of origin and number of animals that were considered for genotyping of *Echinoccocus granulosus* strains.

Origin	Number of animals				Total

	Cattle	Goats	Sheep	Camels	
Nadunget	32	5	4	0	41
Rupa	30	3	2	0	35
Katikekile	15	2	1	0	18
Tapac	8	1	0	1	10
**Total**	**85**	**11**	**7**	**1**	**104**

### DNA extraction

DNA extraction from tissue samples was performed as described by Nakao, Sako and Ito (2003), where small sections of the hydatid cysts or single isolated protoscolices were lysed using 20 µL of 0.02 M NaOH, heated at 99 °C for 10 minutes and used directly as template.

### Nested polymerase chain reaction amplification

First amplification of the NADH dehydrogenase 1 gene (NADH 1 gene) was performed as described by Addy *et al*. (2012) using primers nad A (5’TGT TTT TGA GAT CAG TTC GGT GTG 3’) and nad C (5’CAT AAT CAA ACG GAG TAC GAT TAG 3’) as forward and reverse primers, respectively. A 25-µL reaction mixture containing 2.5 µL of polymerase chain reaction (PCR) buffer (pH 8.3), 2.0 µL of 2 mM MgCl_2_, 0.5 µL of 200 µM of each dNTP, 0.625 µL of 12.5 pmol of each primer, 0.125 µL of 1.25 U of Taq polymerase and 2.0 µL of template DNA were subjected to PCR conditions of denaturation at 94 °C for 5 minutes and then 94 °C for 30 seconds. Annealing was carried out at 55 °C for 30 seconds, elongation at 72 °C for 1 minute, then final elongation at 72 °C for 5 minutes for 35 cycles. The target product size was 1075 base pairs (bp).

A second amplification was carried out under the same conditions using 2.0 µL of amplicon (produced with first amplification) with primers nad B (5’CAG TTC GGT GTG CTT TTG GGT CTG 3’) and nad D (5’GAG TAC GAT TAG TCT CAC ACA GCA 3’) as forward and reverse primers, respectively. The product size for this amplification was 850 bp (Addy *et al*. 2012).

### Restriction fragment length polymorphism

Fifteen microlitres of the nested PCR amplicons was digested using 0.5 µL of Hph I restriction [recognition sequences; 5′…GGTGA (N_8_)*…3′ and 3′…CCACT (N_7_)*…5′] enzyme in presence of 7.5 µL of nuclease-free water and 2.0 µL of 10X buffer B as instructed by the manufacturer (Fermentas, GMBH, Germany). Restriction was performed overnight at 37 °C. The Hph I restriction endonuclease produced a staggered cut at nucleotide positions AT or TA bp 8 bp or 9 bp away from the recognition sequences. *Echinococcus granulosus sensu stricto* (G1–G3) restriction was expected to produce approximately 425 bp, 320 bp and 204 bp, whilst *Echinococcus canadensis* (G6/7) was expected to produce approximately 425 bp and 107 bp following online restriction mapping.

### Agarose gel electrophoresis

Successful amplification of *E. granulosus* NADH 1 gene was determined by electrophoresis on 1.5% agarose gel prepared in 1X tris acetate ethylenediaminetetraacetic acid (TAE) buffer containing 1% ethidium bromide, whilst successful digestion of the NADH 1 gene was electrophoresed on 2.0% agarose prepared in 1X TAE buffer with 1% ethidium bromide. Electrophoresis was performed at 120 V for 45–55 minutes; separated fragments were visualised under UV transillumination.

### Sequencing

Ten percent (*n* = 8) of the samples that produced the G1–G3 banding patterns on restriction fragment length polymorphism (RFLP) and all the samples (*n* = 3) that produced G6/7 patterns were sent for Sanger DNA sequencing assay at Inqaba Biotech Industries Ltd, South Africa.

### Data analysis

Gels were photographed and bands manually analysed against positive and negative controls. Following DNA sequencing, chromatograms were visually analysed using BioEdit Sequence Alignment Editor Software. Genotypes were determined online using the National Centre for Biotechnology Information (NCBI) Blast computer programme for homology (http://www.ncbi.nlm.nih.gov/blast/Blast.cgi) against published sequences in the GenBank. Pairwise sequence alignments were made using the online BLAST programme against reference sequences obtained from the GenBank. Genetic relatedness of the *E. granulosus* genotypes were determined using MEGA6 software. The Muscle programme of this software was used for multiple sequence alignments, trimming was carried out and phylogenetic analysis was accomplished using the phylogeny programme of the same software.

## Results

A total of 2624 samples from ruminant livestock, including cattle, goats, sheep and camels slaughtered at Moroto Municipal Council abattoir, were examined by a Veterinary Officer for the presence of hydatid cysts in visceral organs. Of the examined animals, 104 that had suspected cysts were sampled (Table 1). These samples were subject to PCR-RFLP for genotyping *E. granulosus* strains (Addy *et al*. 2012) and 10% of positive samples for DNA sequencing. A majority, 81.2% (*n* = 85) of the suspected samples, were collected from cattle, 10.6% (*n* = 11) were from goats, 6.7% (*n* = 7) were from sheep, whilst only 0.96% (*n* = 1) was extracted from a camel (Table 1).

### Amplification of *Echinococcus granulosus* NADH 1 gene

**FIGURE 2:**
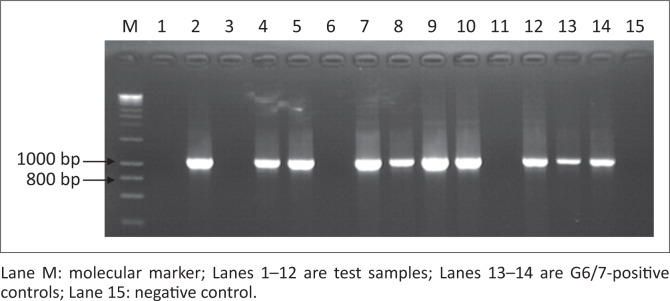
A 1.5% agarose gel showing *Echinococcus granulosus* NADH 1 gene nested PCR analysis results.

Of the suspected 104 samples, successful amplification of the *E. granulosus* NADH 1 gene occurred in 73.1% (*n* = 76) of the samples producing a band of approximately 850 bp, whilst 26.9% (*n* = 28) were not successfully amplified. Figure 2 shows a representative gel for the *E. granulosus* NADH 1 gene PCR products on 1.5% 1X TAE agarose gel electrophoresis.

### Genotyping of *Echinococcus granulosus* strains in Moroto district by restriction fragment length polymorphism

**FIGURE 3:**
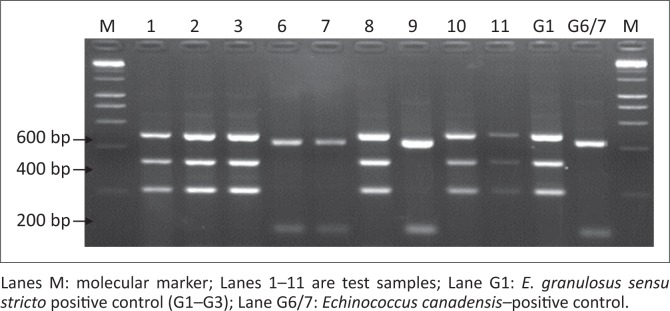
A 2.0% agarose gel showing *Echinococcus granulosus* NADH 1 gene RFLP analysis results.

Of the *E. granulosus* NADH 1 gene successfully amplified samples (*n* = 76), 96.05% (*n* = 73) were genotyped by RFLP as G1–G3, whilst, 3.95% (*n* = 3) were G6/7. A representative gel following restriction of nested PCR product by Hph I was as shown in Figure 3.

### Genotyping of *Echinococcus granulosus* strains in Moroto district by sequencing

Following the inability of RFLP genotyping assay to resolve genotypes G1–G3 and G6/7, 10% of the samples that yielded the G1–G3 (*n* = 8) restriction pattern and all three that produced the G6/7 patterns were subjected to DNA sequencing. Using a BLAST search, sequences were compared with published sequences in the NCBI GenBank (Table 2).

**TABLE 2:** Comparison of test sequences with those from the GenBank.

Number	Sample	RFLP	Sequencing results	Maximum identity (%)	E-value	GenBank accessions	Other accessions
1	MRT3A	G1–G3	G1	99	0	KJ556993	EF367321
2	MRT08	G1–G3	G1	99	0	KJ556993	EF367321
3	MRT60A	G1–G3	G1	99	0	KJ556993	EF367321
4	MRT61	G1–G3	G1	99	0	KJ556993	EF367321
5	MRT66	G1–G3	G1	99	0	KJ556993	EF367321
6	MRT76	G1–G3	G1	99	0	KJ556993	EF367321
7	MRT77	G1–G3	G1	99	0	KJ556993	EF367321
8	MRT78	G1–G3	G1	99	0	KJ556993	EF367321
9	MRT47	G6/7	G6/7	100/99	0	AB208063	AB235847
10	MRT48	G6/7	G6/7	100/99	0	AB208063	AB235847
11	MRT49	G6	G6/7	99	5e^-155^	HM749618	HM749617

RFLP, restriction fragment length polymorphism.

All the samples (*n* = 8) that yielded the G1–G3 pattern were homologous to the common sheep G1 strain with maximum identity of 99%. Meanwhile, 66.7% (*n* = 2) of the samples that produced the G6/7 restriction pattern remained unresolved because they showed homology with both G6 and G7 GenBank reference sequences at 99/100% maximum sequence identity. One (*n* = 1) of the sequences that yielded G6/7 RFLP pattern was homologous at 99% maximum sequence identity to the G6 strain alone, although with a poor *E*-score (4e^-155^).

### Phylogenetic analysis

Of the 11 samples that were subjected to DNA sequencing, 10 produced uniformly sized sequences (≈ 520 bp). Only one (MRT049) produced a shorter sequence (≈ 312 bp); it was not used in MEGA alignment and phylogenetic analysis. Therefore, 10 sequences were considered for comparison with other sequences in the NCBI GenBank. The criterion for the NCBI sequence selection absolutely relied on the genotype and size of the sequences (> 700 bp). Of these, eight sequences belonged to G1 genotype whilst two sequences belonged to G6/7 genotype. Table 3 shows sequences selected from NCBI GenBank for phylogenetic analysis.

**TABLE 3:** Test sequences (MRT) and GenBank accession numbers for sequences used in phylogenetic tree construction.

Accession number	Genotype	Host species	Year of identification	Country of origin	Maximum identity (%)
Chamai *et al*. (MRT03A)	G1	Cattle	2014	Uganda	-
Chamai *et al*. (MRT08)	G1	Cattle	2014	Uganda	-
Chamai *et al*. (MRT060A)	G1	Cattle	2014	Uganda	-
Chamai *et al*. (MRT061)	G1	Cattle	2014	Uganda	-
Chamai *et al*. (MRT066)	G1	Cattle	2014	Uganda	-
Chamai *et al*. (MRT076)	G1	Cattle	2014	Uganda	-
Chamai *et al*. (MRT077)	G1	Cattle	2014	Uganda	-
Chamai *et al*. (MRT078)	G1	Cattle	2014	Uganda	-
Chamai *et al*. (MRT047)	G6/7	Cattle	2014	Uganda	-
Chamai *et al*. (MRT048)	G6/7	Cattle	2014	Uganda	-
EF367332	G1	Sheep	2007	Morocco	99
EF367319	G1	Sheep	2007	Morocco	100
KJ556993	G1	Human	2014	China	100
KC579442	G1	Cattle	2013	Argentina	99
EF090610	G1	Sheep	2006	India	99
AB208063	G6	Camel	2009	Kazakhstan	100
AB235847	G7	Pig	2009	Japan	99
KJ556995	G7	Human	2014	China	99
AB235848	G8	Moose	2009	Japan	97
KJ663949	G10	Human	2014	China	98

**FIGURE 4:**
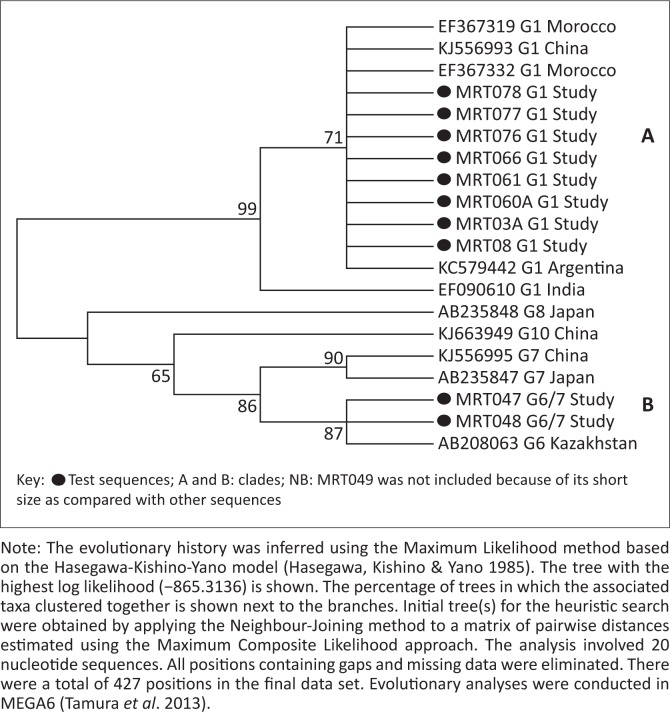
Molecular phylogenetic analysis of test isolates along with National Centre for Biotechnology Information published sequences by Maximum Likelihood method.

Genetic relatedness of test isolates with those in GenBank was assessed using Maximum Likelihood method based on the Hasegawa-Kishino-Yano model (Hasegawa, Kishino & Yano 1985) and revealed two large clades. Clade A comprised G1s in which the G1 isolates from the present study clustered with those of Morocco, China, Argentina and India. Clade B cluster comprised the G6/7 isolates from the present study along with genotypes G6–10 from Japan, China and Kazakhstan (Figure 4). No published GenBank sequences were obtained from East Africa countries including Uganda.

## Discussion

This is the first study to genetically characterise the genotypes of *E. granulosus* amongst livestock in Uganda. Our data clearly showed the predominance of *E. granulosus* genotype G1 (96.05%) corresponding to the common sheep strain, with a minority (3.95%) of cases being *E. canadensis* (G6/7) genotype. None of the other strains reported elsewhere, including the buffalo strain (G3), the equines strain (G4) and the still poorly characterised cervid strains (G8, G9 and G10) were found in this sample from Moroto district. Our findings were in agreement with those of Addy *et al*. (2012), who reported the predominance of genotype G1 (almost 100%) in neighbouring Kenya, but they also reported the occurrence of *E. ortleppi* genotype, not found in our study.

Our findings differed from studies conducted in Sudan, which reported predominance of the camel strain (G6) and to a lesser extent (< 26%), the cattle strain (G5) (Dinkel *et al*. 2004; Omer *et al*. 2010). Some African camel-rearing countries like Algeria, Libya and Tunisia have also reported the existence and predominance of G1 over G6/7 (Abushhewa *et al*. 2010; Farjallah *et al*. 2007; Maillard *et al*. 2007). This could be attributed to continental globalisation of the common sheep strain (G1) from Europe (Spain and Portugal) to northern African countries, especially Libya, Algeria and Morocco, which are geographical neighbours. These reports indicate regional variations of the *E. granulosus* genotypes, although the common sheep strain (G1) appears to be predominant. In Turkey, for example, Snabel *et al*. (2009) also reported the sheep strain (G1) as the predominant genotype.

Our data suggest that the *E. granulosus* genotypes circulating in dogs and livestock in Karamoja region could be originating from the neighbouring Turkana region of Kenya. The borders between these regions are porous, with unrestricted movement of livestock; moreover, the lifestyle of the Karamojong and Turkana could also be a major factor that explains existence and persistence of echinococcosis in these areas. The lifestyle of these pastoralists is mainly characterised by close interaction with dogs, poor hygiene, insufficient veterinary services and lack of knowledge of disease and its mode(s) of transmission. Besides these, unending cattle rustling practices amongst these nomadic groups could also explain the spread of echinococcosis between Karamoja sub-region and Turkana of Kenya.

In agreement with Eryildiz and Sakru (2012), both genotypes (test genotypes) were closely related to other studies. Most of the clade A study Moroto (MRT) G1 strains shared the same node with other strains, indicating a monophyletic relationship. This meant that the G1 genotypes of other studies were homologous with G1s of the study. Similarly, clade B strains, which included the study MRT G6/7, also revealed origin from a common ancestry. Although the study revealed a divergence at the basal nodes with reduced bootstrap values in clade B, this could be explained by earlier claims of poor characterisation in the *E. canadensis* (G6–G10) group, as reported by Boubaker *et al*. (2013). Nevertheless, genotypes G8 and G10 in particular showed greater distinctness from the others, which almost rendered the relationship in clade B paraphyletic. This distinctness could be attributed to the fact that both are cervid strains (wild) as compared with the rest of the strains that are domestic. This did not, however, rule out common ancestry with the rest of clade B genotypes.

The biggest setback to the study was the absence of published eastern Africa GenBank sequences, especially for Kenya and Uganda, because it was believed that Moroto echinococcal disease was shared with Turkana of Kenya, but it was not possible to compare the study findings with other eastern African countries. This therefore presented a missed opportunity for a better understanding of *E. granulosus* genotype distribution in eastern Africa, especially in Uganda (Moroto), Kenya (Turkana) and South Sudan (Toposa). The study was also hindered by the low resolution power of the RFLP assay that failed to distinguish between G1–G3 and G6/7 genotypes, although this was circumvented through DNA sequencing, which was costly.

## Conclusion

The project determined the strains of the dog tapeworm responsible for the echinococcal disease amongst livestock in Moroto district (Karamoja area) to be the common sheep strain (G1) of *Echinococcus sensu stricto* and *E. canadensis* (G6/7). No other strains of *E. granulosus* were identified.

Our finding of predominance of the G1 genotype in Moroto district (Karamoja), a genotype identified as the chief causative agent of hydatidosis across all species, calls for institution of strict public health measures that include meat inspection, proper disposal of cystic organs to avoid access by dogs and restriction of movement of animals from one area to another. Notably, the study needs to be complemented with studies amongst humans and dogs. This will help to map a linkage between the definitive host, the grazing intermediate ungulates and humans who are adversely affected by this infection compared with other intermediate hosts. This will make understanding of the disease epidemiology better in Karamoja, hence better public health mitigation and treatment strategies.
